# Ascertaining Whether an Intelligent Voice Assistant Can Meet Older Adults’ Health-Related Needs in the Context of a Geriatrics 5Ms Framework

**DOI:** 10.1177/23337214231201138

**Published:** 2023-09-30

**Authors:** Ella T. Lifset, Kemeberly Charles, Emilia Farcas, Nadir Weibel, Michael Hogarth, Chen Chen, Janet G. Johnson, Mary Draper, Annie L. Nguyen, Alison A. Moore

**Affiliations:** 1 University of California San Diego, La Jolla, USA; 2 University of Southern California, Alhambra, USA

**Keywords:** technology, multimorbidity, qualitative research, aging

## Abstract

The Geriatrics 5Ms: Medications, Mind, Mobility, what Matters most and Multicomplexity is a framework to address the complex needs of older adults. Intelligent Voice Assistants (IVAs) are increasingly popular and have potential to support health-related needs of older adults. We utilized previously collected qualitative data on older adults’ views of how an IVA may address their health-related needs and ascertained their fit into the Geriatrics 5Ms framework. The codes describing health challenges and potential IVA solutions fit the framework: (1) Medications: difficulty remembering medications. Solution: reminders. (2) Mind: isolation, anxiety, memory loss. Solution: companionship, memory aids. (3) Mobility: barriers to exercise. Solution: incentives, exercise ideas. (4) Matters most: eating healthy foods. Solution: suggest and order nutritious foods, (5) Multicomplexity; managing multimorbidity. Solution: symptom tracking and communicating with health care professionals. Incorporating the 5Ms framework into IVA design can aid in addressing health care priorities of older adults.

## Introduction

Older adults are the fastest growing group of digital technology users (Anderson & Perrin, 2017), but often such technologies fail them because they are not usable or useful (Corbett et al., 2021; Kowalski et al., 2019; Schlomann et al., 2021). Intelligent Voice Assistants (IVAs) are embodied in smart speakers such as Amazon Echo^®^ and Google Home^®^, and are present in almost a quarter of US households (Richter, 2020). Because they do not require visual or tactile interaction, they offer options for accessibility beyond computers and touchscreen devices that may be particularly useful for people with sensory, physical, and cognitive impairments that are common among aging populations (Nallam et al., 2020; Pradhan et al., 2018). Because of the prevalence of IVAs in use and their promise for use among older adults (Stigall et al., 2019), as well as increased isolation and reduced access to health care, which has been pronounced among older adults since the start of the COVID-19 pandemic (Bastani et al., 2021), academia and industry are increasingly examining how IVAs are being used and their potential among older adults, including for health-related needs (Arnold et al., 2022; Chen, Johnson et al., 2021; O'Brien et al., 2020, 2022; Pradhan et al., 2020; Trajkova & Martin-Hammond, 2020).

### The Geriatrics 5Ms

**Figure 1.**
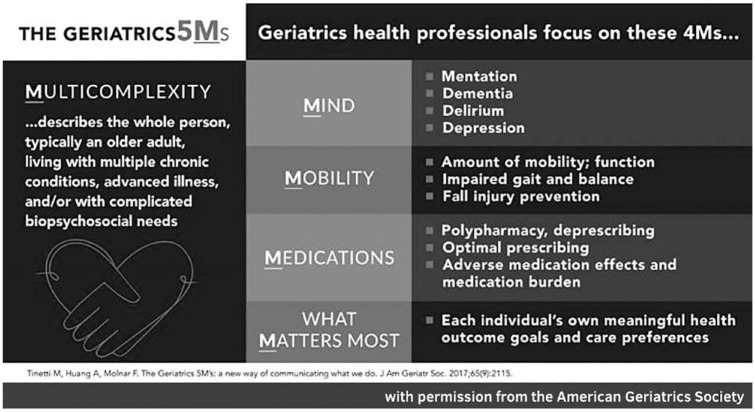
The geriatrics 5Ms.

With advancing age, older adults may develop combinations of diseases, health conditions and disabilities. They often have more complex health care needs and changing health care priorities. Geriatrics healthcare professionals, including geriatricians and others who have advanced training in the care of older adults, comprehensively address the needs of older adults. These healthcare professionals focus on five core areas, known as the Geriatrics 5Ms: Medications, Mind, Mobility, what Matters most and Multicomplexity. The 5Ms represent domains that are important to care for people as they age and were developed in 2017 by Canadian and US specialists in geriatric medicine (Tinetti et al., 2017). *Medications* includes optimal prescribing, adherence to prescribed medications and reducing unnecessary or harmful medications. *Mind* includes addressing mentation or cognition, dementia, delirium and depression. *Mobility* includes amount and quality of mobility, support for safe mobility and fall prevention. What *Matters most* focuses on each person’s own health outcome goals and care preferences. *Multicomplexity* describes the whole person, for example, an older person living with multiple diseases and conditions and having complicated needs (Figure 1).

This framework both communicates the expertise of geriatrics healthcare professionals and organizes common health-related concerns of older adults into the five core areas. Having such a framework assists health care providers in identifying, assessing and addressing health-related needs of older adults. The Geriatrics 5Ms framework is related to the Age-Friendly Health System initiative (Fulmer et al., 2018) whose goals are to reduce health care-related harms to older adults, improve care and satisfaction with care, and optimize value for all. This initiative, developed between 2015 and 2017, includes 4Ms: Mind, Mobility, Medications and what Matters (K. S. Mate et al., 2018). Since then, the evidence supporting this framework in improving care for older adults was reviewed and summarized by an expert panel and found to be robust (K. Mate et al., 2021). These two frameworks, the 5Ms and the 4Ms, may be used by all medical disciplines and specialties, can be applied to any older person, and utilized in any site of care.

### Interactive Voice Assistants for Older Adults’ Health Care Needs

IVAs may offer a way to address older adults’ health care needs as outlined in the 5Ms framework. Though there is a growing literature on the subject of IVAs and older adults (Arnold et al., 2022; Even et al., 2022; Jakob, 2022), there is a more limited literature on designing and utilizing IVAs from an older adult health-related needs perspective, and none of these studies have utilized the comprehensive 5Ms framework (Arnold et al., 2022; Chen, Mrini et al., 2021; Jesús-Azabal et al., 2020; Nallam et al., 2020; O'Brien et al., 2020, 2022; Pradhan et al., 2018; Ruggiano et al., 2021; Shade et al., 2020).

To explore the potential for IVAs to assist with health care needs for older adults, a prior study interviewed older adults with varying experience levels with IVAs (Chen, Johnson et al., 2021). The interviews included questions about (1) medication and healthcare management approaches and challenges, (2) communication with healthcare providers, (3) daily routines and challenges, and (4) how IVAs were used and features that would be desirable to address health care needs. Data were coded using descriptive coding to extract summaries of the ideas present in the text. The main themes that arose were: (1) challenges in managing medications and adhering to medication schedules, (2) experiences of loneliness and challenges interacting with other people during the COVID pandemic, (3) difficulty in monitoring and reporting health and activities to health care providers and caregivers; (4) challenges remembering appointments with health care providers, (5) frustration with technology and challenges interacting with IVAs due to hearing impairment and problems with speech recognition, (6) gaps in IVA features used and those desired, and (7) concerns about data privacy with IVAs.

No prior studies have examined whether older adults’ health care needs and potential for an IVA to address them have utilized the Geriatrics 5Ms framework, therefore we sought to contribute to the literature by examining if the qualitative codes that emerged in the prior study (Chen, Johnson et al., 2021) might be placed within the 5Ms framework. If so, then one might design IVAs to address health care needs of older adults based on the Geriatrics 5Ms with the potential to both address health care priorities of older adults and improve the quality of their health.

## Methods

### Overview

This study analyzed qualitative data collected through individual participant interviews in a prior study (Chen, Johnson et al., 2021) whose aims were to identify barriers and opportunities for IVA use by older adults with a focus on health-related needs. In the following sections, we summarize the original study procedures as well as our analytical approach for this current study.

### Recruitment

Participants were recruited through flyers posted in the waiting room of a Geriatric Medicine Clinic in San Diego, California. Inclusion criteria were age ≥65 years and English-language proficiency. A $20 gift card was provided as an incentive for participation. The study was approved by the university’s Human Research Protections Program. Each participant was asked to consent to the interview and its recording prior to participation.

### Participant Characteristics

Sixteen individuals completed the interviews. Their ages ranged from 68 to 90 years. Most were White (*n* = 12), just over half were men (*n* = 7), two were Asian, one was Black, and one was Hispanic. Fifteen had at least some college education and one had high school education. One-third of the participants lived in retirement communities (*n* = 5). Over half of the participants had some prior experience with IVAs (*n* = 9), with a range of little to extensive experience with this type of technology.

### Data Collection

Because the study occurred during the COVID-19 pandemic, interviews were conducted via telephone and audio-recorded. The semi-structured interviews lasted up to 1 hour, using a question guide developed based on prior interviews with a sample of the clinic’s health care providers, the expertise of the study team, and relevant literature (Chen, Johnson et al., 2021).

### Data Analysis

Audio files were transcribed verbatim using Amazon Transcribe software (https://aws.amazon.com/transcribe/) and manually checked for errors. The corrected transcriptions were then de-identified, coded and interpreted through an inductive and deductive grounded theory approach (Elo & Kyngäs, 2008; Strauss & Corbin, 1994). Optimal Workshop software (www.optimalworkshop.com) was used to facilitate coding.

For this study, the original descriptive codes were extracted and two reviewers (de-identified for review purposes) independently assigned each code into categories corresponding to the 5Ms (Medication, Mentation, Mobility, Matters most and Multicomplexity) through an axial coding exercise. Representative quotes were selected to illustrate each category and analyses proceeded in an iterative manner. The reviewers held consensus discussions to clarify disagreements and discrepancies were discussed until consensus was reached.

## Results

### Distribution of Codes Into the 5Ms

**Figure 2.**
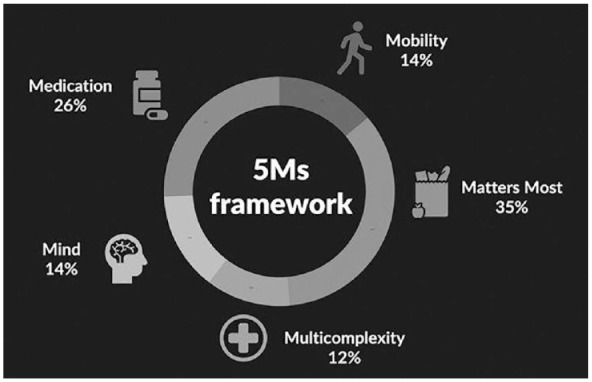
5Ms framework. *Note*. Percentages of codes categorized into each of the 5Ms.

From the original data, 111 codes were utilized; 21 codes were omitted as they did not focus on health. The codes were sorted into each of the five categories. The largest number of codes was in the category of Matters most (*n* = 39), followed by Medication (*n* = 29), Mind (*n* = 15), Mobility (*n* = 15) and Multicomplexity (*n* = 13) (Figure 2).

### Medication

All of the participants discussed the category of Medication in their interviews. Most reported challenges with medication management.

*“Well, I’m fine as long as I have to take in the morning and take at night, you know? But it’s when they say okay, you know, with carbamazepine you’re gonna have to take some at noon time, too, and then to remember to have the bottle out. So that’s one that gets confusing for me. . .”*

*“Because if we rush and we get there, late then, you know, we fix breakfast with that and then we forget that we have to give him the medication. I’ve gotten into the habit when I’m setting the table, I make it part of setting the table–the little box. But I don’t just put the box. I put the pills on a napkin so that he sees it. So that has been part of my setting the table.”*

Here, both participants report difficulty in adhering to medication schedules. These comments also highlight an awareness that the participants have regarding the disadvantages in their reminder methods.

Commonly, participants highlighted medication reminders as a potential use for an IVA.

*“I take a bunch of vitamins. I think that’s one of the things that would be good to tell. It reminds us. Did you take your medication? . . . It’s 9:00 it’s time to take your medication because I get distracted very easily and I start doing things. . .But if it was a voice saying hey, there’s a medication, time for a medication then that would be that would be good.”*

An IVA that provided reminders and other cues to support medication adherence could potentially address these concerns.

Other participants mentioned adverse effects from medications and also taking supplements.

*“. . .when I took gabapentin, they had me taking such a large dose. . . . I told them I couldn’t take it anymore because I was having suicidal ideas. . . and I was like wow, where’s this coming from? And I didn’t believe it when people said that this could happen to you if you take this med or you take that med. Yeah, you got to be careful because I was a cooperative person. Just like that. Go suicidal, you know.”*

*“..my wife is quite active on the different health blogs on the internet. She will come up with different supplements that I’m supposed to take and I take 'em. And then she goes off them and I don’t. I just keeping adding to my list.”*

An IVA could provide information on potential adverse effects from prescription, and non-prescription medications, as well as supplements. To improve safety, an IVA could also provide evidence-based information on potential interactions of supplements with medications as well as medication interactions.

### Mind

Ten of the participants (63%) discussed the category of mind in their interviews. Participants mentioned isolation, depression and anxiety as substantial health concerns during the interviews, which were magnified by the global COVID-19 pandemic.

*“Oh, isolation. Um, I normally have some difficulty with depression, uh, and anxiety. So I would say that it’s bumped it up a notch and that also, I’ve had big problems all my life with insomnia and sleep patterns. And I would say that it’s bumped it up a notch to where the difficulty [is] getting to sleep. . .”*

*“And I think actually, the COVID thing has got me a little bit depressed. . . ..I don’t have too many people calling me”*

IVAs may provide companionship through personalized interactions or access to programs to help to reduce feelings of loneliness, which could potentially address these concerns.

The inability to recall appointments or to schedule them for the future was also mentioned. Here the respondents refer to a need for a better reminder system for medical appointments:

*“Well, I have a calendar on my computer, and, um and I also have paper copies of things like a file that I could look in that I do. And I also have little, uh, calling cards, business card types of things that I have on my desk. Tell me what’s coming up. So, I have three different ways that I keep track. . .”*

*“I kept on telling him no it’s on Monday, and he said, ‘No, it’s on Tuesday.’ Then I go, ‘Check [medical portal de-identified for review purposes] and you’ll see.” And so he went. And I was right. So, you know, that kind of thing is always helpful. If you can say, ‘Hey, Alexa, when is my next appointment? You know?’ And then, uh, it would look at my calendar and tell me, ‘Oh, your next appointment is with so and so on a date.’ So that would [be a] big help”*

Here, the first participant reports a convoluted reminder system for health care appointments. This is a clear potential use for an IVA–a method to schedule events via an intuitive, voiced-based approach that could assist with scheduling appointments and appointment reminders. This is similar to what the second participant proposes; a system that could tell the user when his or her next health care appointment is. However, this could be taken a step further, with an IVA that could tell the user when his or her appointment is, and schedule future appointments with the user’s health care provider.

### Mobility

All but one of the participants (94%) discussed topics directly related to the category of Mobility in their interviews. Participants identified reminders to move as a need, as well as a lack of variety in exercise as a concern.

Below, the participant illustrates a desire to stay active, through scheduled walks:

*“So anyway, or actually, I’m gonna have to put a clock out there where I can see it and remind me to get up and move. You know, I have a tendency to sit in the recliner too long. Alexa could be telling me my time for [my] four o’clock walk”*

Here, the participant wants to avoid being sedentary. However, the participant also reports her inability to regularly remember to exercise, and expresses a desire for a voice-activated program that will help her stay mobile.

*“Well, I wish. . . that I could have the discipline, you know, to do some more stretching. I don’t want to wind up like I was before, stuck in the chair, you know, and having another heart attack”*

This participant expresses her desire to exercise with others and to engage in stretching activities, but reports she is unable to do so due to her self-described lack of discipline. This participant wants to avoid complications of being sedentary, but does not have the tools to facilitate increased activity. An IVA could not only provide reminders to exercise, but could also collect health data, such as exercise time, which could be shared to help monitor progress and provide encouragement.

In addition, there were several responses indicating activity restrictions, which could limit mobility and adversely affect health.

*“There’s not very much for us to be doing except our daily walk”*

*“Once upon a time, um, I exercised on some of the machines here. I swam every day. Now I swim, but not every day [until] I broke my elbow at the end of April, which kind of hampered my activities”*

*“I do walk the hallway if somebody’s with me, I don’t want a fall or anything, but, um, yeah, with COVID. . .we’ve been closed in more. I watch more TV”*

The first participant states that there is nothing for her to do, except for walking. The second participant states that after sustaining an injury, she is unsure of how to proceed with activity, while the third states that the COVID-19 pandemic has limited her ability to move about and exercise–resulting in a more sedentary lifestyle for each of them.

Another participant noted that technology can help remind one to move.

*“I know my daughter, for example, she set her, I think her phone for every 30 minutes or so. So she doesn’t just sit there ..so she doesn’t forget to get up and walk around. Technology can remind us if we want to use it that way. And if it’s available”*

An IVA might provide exercise options unique to each person. For example, among the participants quoted here, when prompted, the IVA could recommend different walking paths for the first participant, exercises avoiding the use of the elbow or strengthening exercises for that muscle group for the second participant, or home exercises for the third participant.

### Matters Most

All participants discussed topics related to the category of Matters most. Participants mentioned facets of life that “matter most” to them, but did not necessarily have the tools to optimize and some had ideas for how an IVA could assist them with various tasks.

*“I talk to a nutritionist, I have a nutritionist that calls me once a month. I’ve changed my eating habits and eat a lot more vegetables. A lot more vegetables at every opportunity. I try to eat not so much meat, but take care. . .”*

*“It could help me with recipes. Yes. When I’m cooking, I’m trying to cook very healthy.”*

Several participants had similar statements–aspects of daily life about which they have a particular concern. Here, the first participant mentions nutrition as something important to him. For something that can be tracked, such as food intake, an IVA could be designed to allow for user input, track the data and, for example, send it to the person’s primary health care provider, their family members, friends or be used exclusively for the participant’s benefit. In the case of this participant, a tracking system could assist him in maintaining proper nutrition–limiting meat, while eating more vegetables, for example. The second participant suggested that an IVA could assist with finding recipes to help her provide healthy meals.

Another common topic observed in the “Matters most” category was independence and independent living:

*“So now I pay all my bills online, which I couldn’t have done before. And this year, I didn’t know how to do my own taxes online. And with my friends help, we got it done. So, I have a long way to catch up. The older you get you just see things speeding past you, you know?”*

*“Okay, I live by myself. I live in a retirement community, uh, independent living. And I’m 90 years old, I turned 90 in June”*

In these examples, the participants highlight the importance of independence and independent living. The first participant describes her desire to stay financially independent, as well as a willingness to learn; something shared across several of the interviews. For the second participant, it is a source of pride that she is living without assistance. IVAs could potentially support independence through connections with programs to assist with taxes or other services such as food delivery.

### Multicomplexity

Thirteen participants (81%) mentioned items in the theme of multicomplexity. Multicomplexity issues that came up most often included challenges with tracking and then transferring information to an online medical portal, staying in contact with friends and family, as well as need for a symptom checker–all while dealing with multiple illnesses and complicated health needs.

*“. . .I have to weigh myself every day and take my blood pressure and my sugar and keep all that down. And, uh, my friend said that would be a great thing for me to be able to have Alexa keep that information and then just be able to transfer to [medical portal de-identified for review purposes], You know, instead of me having to type it all in for the doctors. Yeah. I’ll write it down but then I will miss three days of writing things down just because I get distracted and doing something else, you know”*

Older adults value a way to track and report health data directly to their primary care providers that medical portals do not facilitate. An IVA that allows for health data tracking and data sharing with health care providers and others could address these needs, especially those living with multiple conditions.

Maintaining contact with others despite dealing with multiple conditions was also mentioned.

*“First time I came down with MRSA, or staph, was after I got out of surgery. . ., I ended up staying in the hospital for over a month and then having to get treatment. And a treatment stayed in my arm for much . . .longer than that. . . . But, uh, sometimes that impeded my ability to meet with people the way I wanted to. . .”*

An IVA could potentially reduce the burden that a patient struggling with multiple illnesses might experience in maintaining relationships. For instance, a patient could utilize the IVA to update a family member about a treatment received, or stay in contact with a care partner.

Finally, due to the complexity of their conditions, patients with multiple illnesses might also need a way to track health-related parameters.

*“. . .it’s just a routine check that they have to do every year. And to see, you know, if I have symptoms, which I don’t, and to see how my lymphoma, the cancer cells, the lymphocytes are acting up”*

**Figure 3.**
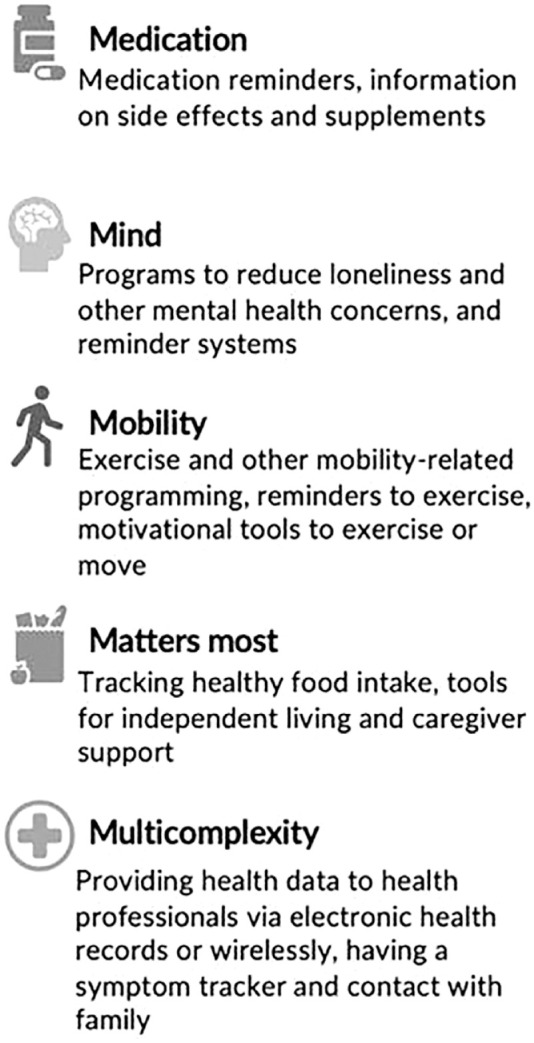
Older adult-informed health-related uses for an intelligen voice assistant.

An IVA could track symptoms longitudinally, which could be exceptionally helpful for those living with multiple chronic illnesses. It could allow the patient to better keep track of when she or he experienced certain symptoms over a prolonged period. This could be helpful for the provider as well–these results could be accessed by the health care provider and could lead to better understanding of disease processes and/or more effective treatments. A summary of our findings for health-related uses for IVA is presented in Figure 3.

## Discussion

Overall, we found that older adult participants’ needs for an IVA related to health can be conceptualized through the Geriatrics 5Ms framework. Using older adults’ interview data from a prior study, we identified numerous uses for an IVA that may not have been identified had we not utilized this framework focused on the needs of older adults. In the Medication category, older adults reported challenges with medication adherence and concern about adverse effects. Solutions including medication reminders and provision of comprehensive evidence-based information on medications were highlighted as a potential IVA use. In the Mind category, isolation, participants identified isolation, depression and anxiety as well as difficulty remembering appointments as concerns. IVAs might assist with mental health challenges via personalized companionship or connection to programs to mitigate mental health challenges. IVAs also have the potential to issue reminders for appointments. In the Mobility category, reminders to move and variety in exercises were identified as potential IVA uses. IVAs could also share progress with exercise time and intensity with users and potentially share these data with others including health care providers to enhance motivation and accountability. In the Matters most category, older adults’ desired assistance with various tasks or activities related to health and independence such as monitoring nutrition, cooking healthy meals and paying taxes. IVAs could assist with tracking food intake that could be shared with others, providing healthy recipes, or through connecting to programs to support independence such as tax preparation or food delivery. Finally, in the Multicomplexity category, participants reported challenges with tracking and sharing health data with their health care providers’ portals and connecting with others when facing complex illnesses. IVAs might address these challenges by tracking and sharing user data with online medical portals, maintaining contact with loved ones.

No prior studies have examined whether older adults’ health care needs and potential for an IVA to address them have utilized the Geriatrics 5Ms framework, therefore we sought to contribute to the literature by examining if the qualitative codes that emerged in the prior study (Chen, Johnson et al., 2021) might be placed within the 5Ms framework. If so, then one might design IVAs to address health care needs of older adults based on the Geriatrics 5Ms with the potential to both address health care priorities of older adults and improve the quality of their health.

While this study focused on whether older adults’ health care needs and potential to address them using an IVA could utilize the Geriatrics 5Ms framework rather than the feasibility of proposed solutions, we would like to comment on how currently available IVAs might address older adults’ health care needs and where there is need for improvement. Many of the proposed solutions are currently feasible using an IVA. For instance, reminders with set parameters [(e.g., at particular times of day (8 am and 5 pm), or frequencies (monthly)] are available and may be used for medication, appointments, exercise prompting and more. Additionally, IVAs have the capacity to make suggestions in response to a request (e.g., personalized healthy recipes, specific medication interactions). IVAs have the capability to contact others (e.g., when facing complex illnesses or to mitigate loneliness). It is important to consider that several of the solutions proposed to address the needs identified using the 5Ms framework would require third party applications to complete the identified task (e.g., weight or nutrition monitoring). There are also tasks that commercially available IVAs are not yet able to complete (e.g., personalized companionship beyond the pre-programed responses to personal questions, track and share longitudinal exercise and health data for both users and providers).

Most of the literature on the subject of IVAs for older adults has focused on usability and acceptance (Arnold et al., 2022; Even et al., 2022; O'Brien et al., 2020, 2022; Pradhan, 2020). Overall, evidence suggests that there are more benefits than challenges for older adults using IVAs including increased accessibility compared to other computing devices, and increased sense of companionship. Challenges included device set up, learning the proper wording to control the device and devices timing out before completion of voice commands. Providing training for optimal use of IVAs has been suggested as a means of addressing variable digital literacy among users and there undoubtedly will be challenges particularly for more complex tasks such as sharing data with health systems. Despite these challenges, these studies demonstrate increased user confidence over time, thus supporting strong potential for adoption of IVAs as a tool for facilitating independence in older adults.

Two scoping reviews of IVA use by older adults identified ways in which IVAs have been used or have the potential to be used by older adults. Arnold et al. (2022) reported that older adults most commonly used IVAs for reminders, alarms and timers, searching the internet for general information, checking the weather and listening to music. Even et al. (2022) found that using the IVA for internet searches and information access was most commonly reported. These authors also examined dimensions of quality of life affected by IVAs for healthy older adults and those with intellectual disabilities and found that a major benefit of IVAs was a feeling of companionship for healthy older adults and a higher perception of independence for those with intellectual disabilities.

Some have examined specific ways in which an IVA may address needs of older adults, including health (e.g., asking the IVA to read to those with visual impairment, supporting wellbeing with healing music, or tracking health indicators) (McCloud et al., 2022; Trajkova & Martin-Hammond, 2020). Though studies have identified needs and/or reported on applications using IVAs that focus on one of more of the 5Ms [e.g., Medications (Azevedo et al., 2018), Mind (Kim, 2021; Rampioni et al., 2021; Ruggiano et al., 2021), Mobility (Luo et al., 2021)], none of them have utilized the comprehensive Geriatrics 5Ms framework to examine how IVAs might assist older adults with common health-related needs.

### Limitations

It is important to note our study’s limitations. Because we analyzed existing data, we were limited by the questions asked in the original study. We are likely to have learned more if we had asked questions about how an IVA might help address health-related needs specifically in the context of the 5Ms framework. Further, the sample was small and recruited from a single clinic and therefore, we may well have obtained a greater range and depth of responses if we had a larger and more diverse sample of participants. Such a sample would provide additional insights regarding how an IVA could meet their health-related needs. Finally, in-person interviewing could not be conducted due to the data collection occurring during the COVID-19 pandemic. Despite these limitations, the analyses demonstrate that the 5M framework provides a reasonable approach to contextualize older adults’ experiences with health and IVAs.

## Conclusion

We propose designing IVAs utilizing the 5Ms framework for the oftentimes complex health-related needs of older adults. Designs incorporating this framework have potential to improve the ability of IVAs to address health care priorities of older adults and their care partners. Utilizing such a framework, in addition to including older adults and those with expertise in geriatrics as part of design teams, will enhance the usability, utility and privacy of IVAs and other technologies for older adults and those who care for them.
